# Analysis of *Acinetobacter* P-type type IV secretion system-encoding plasmid diversity uncovers extensive secretion system conservation and diverse antibiotic resistance determinants

**DOI:** 10.1128/aac.01038-24

**Published:** 2024-11-04

**Authors:** Mosopefoluwa T. Oke, Kailey Martz, Mădălina Mocăniță, Sara Knezevic, Vanessa M. D'Costa

**Affiliations:** 1 Department of Biochemistry, Microbiology and Immunology, University of Ottawa, Ottawa, Ontario, Canada; 2 Centre for Infection, Immunity and Inflammation, University of Ottawa, Ottawa, Ontario, Canada; Universita degli studi di roma La Sapienza, Rome, Italy

**Keywords:** *Acinetobacter baumannii*, bacterial pathogen, secretion system, antibiotic resistance, carbapenem resistance, horizontal gene transfer

## Abstract

*Acinetobacter baumannii* is globally recognized as a multi-drug-resistant pathogen of critical concern due to its capacity for horizontal gene transfer and resistance to antibiotics. Phylogenetically diverse *Acinetobacter* species mediate human infection, including many considered as important emerging pathogens. While globally recognized as a pathogen of concern, pathogenesis mechanisms are poorly understood. P-type type IV secretion systems (T4SSs) represent important drivers of pathogen evolution, responsible for horizontal gene transfer and secretion of proteins that mediate host-pathogen interactions, contributing to pathogen survival, antibiotic resistance, virulence, and biofilm formation. Genes encoding a P-type T4SS were previously identified on plasmids harboring the carbapenemase gene *bla_NDM-1_* in several clinically problematic *Acinetobacter*; however, their prevalence among the genus, geographical distribution, the conservation of T4SS proteins, and full capacity for resistance genes remain unclear. Using systematic analyses, we show that these plasmids belong to a group of 53 P-type T4SS-encoding plasmids in 20 established *Acinetobacter* species, the majority of clinical relevance, including diverse *A. baumannii* sequence types and one strain of *Providencia rettgeri*. The strains were globally distributed in 14 countries spanning five continents, and the conjugative operon’s T4SS proteins were highly conserved in most plasmids. A high proportion of plasmids harbored resistance genes, with 17 different genes spanning seven drug classes. Collectively, this demonstrates that P-type T4SS-encoding plasmids are more widespread among the *Acinetobacter* genus than previously anticipated, including strains of both clinical and environmental importance. This research provides insight into the spread of resistance genes among *Acinetobacter* and highlights a group of plasmids of importance for future surveillance.

## INTRODUCTION

The multi-drug-resistant (MDR) bacterial pathogen *Acinetobacter baumannii* is recognized by both the World Health Organization (WHO) and Centers for Disease Control and Prevention (CDC) as a clinical pathogen of critical concern. Especially problematic in hospital-associated settings, this Gram-negative pathogen is responsible for a broad array of infections, including respiratory tract, bloodstream, urinary tract, soft tissue, and wound infections (1, 2). *A. baumannii* can survive clinical disinfection protocols and is able to persist on abiotic surfaces for over a month, factors that promote its spread in healthcare settings (3, 4). Nosocomial *A. baumannii* is often prevalent in intensive care units (ICUs) and burn wards (3), and mortality rates associated with antibiotic-resistant *A. baumannii* can range from 24% to 83% (5). Community-acquired *A. baumannii* infection rates have also increased in recent decades (1, 6), with antibiotic-resistant and/or hypervirulent strains demonstrating the potential to cause extreme disease and mortality rates up to 64% (2, 7–9). In addition, the spread of resistance to front-line antibiotics such as carbapenems (1, 2) and the emergence of extensively drug-resistant (XDR) and pan-drug-resistant strains (10, 11) have presented global challenges from a clinical perspective.

*A. baumannii* belongs to a phylogenetically diverse genus comprising over 70 established species (12) that can be found in soil and water ecosystems and associated with humans, animals, plants, and insects (13–19). It is classified as a member of the clinically important pathogen group termed the *Acinetobacter calcoaceticus-baumannii* (ACB) complex, which also includes *Acinetobacter calcoaceticus, Acinetobacter nosocomialis, Acinetobacter pittii, Acinetobacter seifertii,* and *Acinetobacter lactucae* (12, 20, 21). Outside the ACB complex, diverse species of *Acinetobacter* are associated with human infections, including *Acinetobacter haemolyticus, Acinetobacter junni, Acinetobacter johnsonii, Acinetobacter soli, Acinetobacter lwoffi*, *Acinetobacter ursingii, Acinetobacter schindleri*, *Acinetobacter bereziniae*, and *Acinetobacter radioresistens* (13, 22, 23). Among these, several species are considered to be emerging human pathogens (24–26). From agricultural and environmental perspectives, genetically diverse *Acinetobacter* species are associated with infections in animals and fish (27–30). Given a greater appreciation for a One Health perspective to understanding bacterial disease, environmental and agricultural surveillance efforts are increasingly highlighting that environmentally derived *Acinetobacter* also represents an important reservoir for antibiotic resistance determinants (27, 28, 31, 32).

P-type type IV secretion systems (T4SSs) represent important drivers of both pathogen evolution and microbial symbiosis, responsible for the transfer of genetic material via conjugation and the secretion of proteins that can mediate host-microbe interactions (33). With respect to horizontal gene transfer, conjugation promotes the spread of genes that contribute to virulence, pathogen survival, antibiotic resistance, and biofilm synthesis (34). In the context of protein secretion, P-type T4SS virulence factor secretion mediates diverse host-pathogen interactions to promote pathogen success during infection (35–37).

Genes encoding a P-type T4SS were previously identified on eight plasmids in clinically problematic *Acinetobacter* strains, including *A. baumannii*, isolated from several countries in Asia (38), and members of this plasmid family have been shown to be transmissible (38–46). The MDR plasmids, harboring the antibiotic resistance genes *bla_NDM-1_* and *aph(3')-VI*, conferred resistance to clinically important carbapenem antibiotics such as imipenem and meropenem and the aminoglycoside amikacin (38), respectively. However, the prevalence of these plasmids among the *Acinetobacter* genus, their associated geographical distribution and genetic diversity, and the level of conservation of T4SS proteins have yet to be fully elucidated. Here, we performed a systematic analysis of P-type T4SS-encoding plasmids in *Acinetobacter*. We identified a group of 53 P-type T4SS-encoding plasmids spread globally among diverse *Acinetobacter* species and one strain of *Providencia rettgeri.* We observed both an exceptionally high level of conservation at the level of T4SS-encoding proteins and an unexpectedly high diversity of antibiotic resistance genes harbored within.

## RESULTS

### Geographical distribution and host strain diversity of P-type T4SS-encoding plasmids

**Fig 1:**
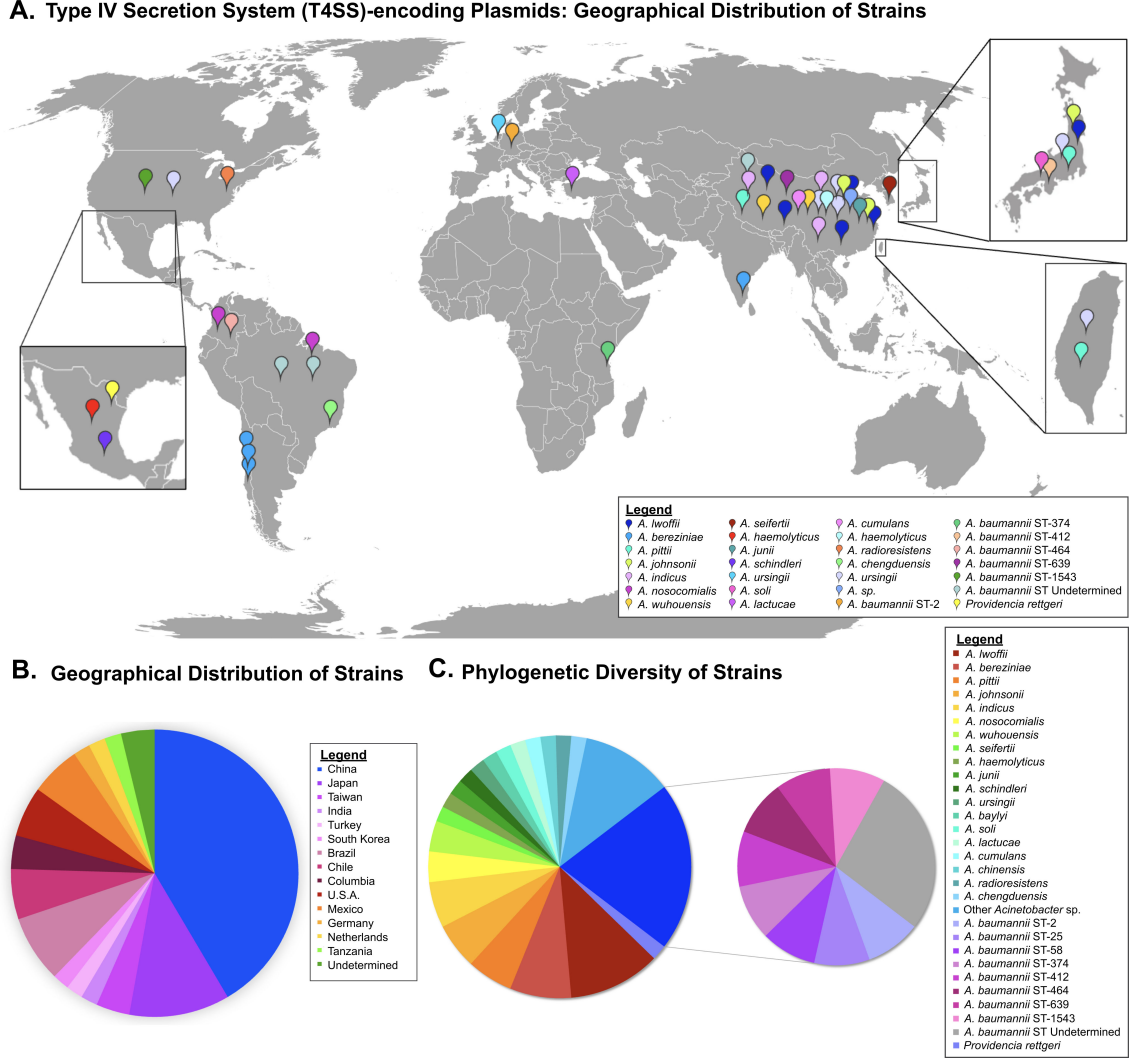
Global geographical distribution and genetic diversity of host strains harboring P-type T4SS-encoding plasmids. Strains without documented information were excluded. (**A**) Geographical distribution of host strains. Pegs denote the country of origin for each strain, and color indicates the species of bacteria. For *A. baumannii* strains, the multilocus sequence type (Pasteur) is shown. (**B**) Country of origin of strains, shown as a pie chart. (**C**) Phylogenetic classification of isolates, shown as a pie chart.

To investigate the geographical distribution and genetic diversity of the P-type T4SS-encoding plasmids, the NCBI nucleotide database was assessed by blast analysis using the P-type conjugative operon from pM131_NDM1 as a query (38). From this analysis, 53 plasmids were identified in diverse species of *Acinetobacter* and one strain of *Providencia* (Table S1). With respect to geographical distribution, the plasmids identified were isolated from 14 different countries across five continents (Fig. 1A and B; Table S1). Over half of the plasmids originated in Asia (62%), consistent with the original publication describing this group of plasmids (38). In addition, 17% of strains were isolated from South America, 11% were from North America, 4% originated in Europe, and 2% were isolated in Africa (Fig. 1A and B; Table S1).

With respect to host range, the P-type T4SS-encoding plasmids were identified in diverse *Acinetobacter* species, including *A. baumannii* (Fig. 1C; Table S1), consistent with previous research (38, 47). However, plasmids were identified in a broader range of *Acinetobacter* and more sequence types among *A. baumannii* than previously reported. *A. baumannii* represented 21% of strains, spanning eight established multilocus sequence types (MLSTs) (Pasteur) (48) (ST-2, 25, 58, 374, 412, 464, 639, and 1,543) (Fig. 1C; Table S1). Of importance, 77% of plasmids were identified in more genetically divergent *Acinetobacter* species (Fig. 1C; Table S1). Among the 20 established species of *Acinetobacter* identified, this included strains within the ACB complex (*A. baumannii*, *A. pittii, A. nosocomialis,* and *A. seifertii*) (20, 21), strains outside the ACB complex that phylogenetically group within the same clade (*Acinetobacter baylyi*, *A. soli*, and *A. ursingii*) (13, 49)*,* as well as more genetically divergent *Acinetobacter* (*A. johnsonii*, *A. schindleri*, *Acinetobacter wuhouensis*, and *A. radioresistens*) (13, 49) (Fig. 1C; Table S1). Of special note, one plasmid was identified outside the *Acinetobacter* genus in a clinical strain of *Providencia rettgeri* (Fig. 1C; Table S1).

In the context of strain origin, 60% of strains were isolated from humans and 25% were derived from environmental locations such as water-associated ecosystems (Table S1). In addition, 6% of strains were derived from cows, 4% were from chickens, and 2% were isolated from pigs (Table S1). With respect to strains of environmental origin, T4SS-encoding plasmids were identified in a broader range of environmental *Acinetobacter* species than anticipated, including species not previously linked to P-type T4SSs (*A. johnsonii* and *A. wuhouensis*) (Table S1). The majority of strains with documented information were isolated after 2010, with 91% of strains derived within this isolation span (Table S1).

Collectively, this demonstrates that P-type T4SS-encoding plasmids are harbored in more genetically diverse strains of *Acinetobacter* than previously anticipated and that the strains are widespread in human and non-human hosts, as well as in the environment.

### P-type T4SS-encoding plasmids: genetic diversity

**Fig 2:**
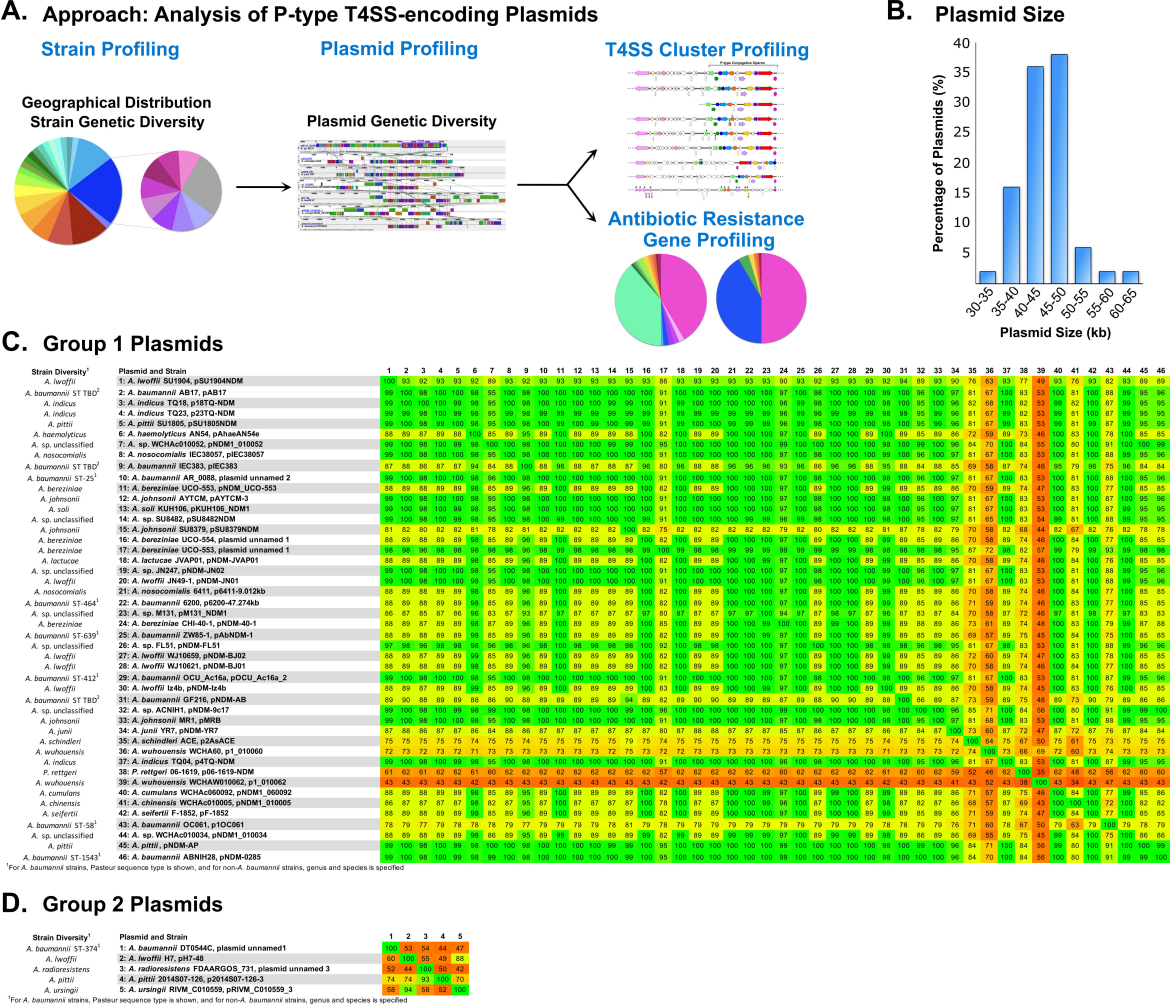
Genetic diversity of P-type T4SS-encoding plasmids. (**A**) Schematic diagram of approach for the systematic study of P-type T4SS-encoding plasmids. (**B**) Plasmid size, shown as a bar graph with 5-kb intervals. (**C and D**) Phylogenomic analyses of P-type T4SS-encoding plasmids plotted as heat maps. The colors are shown from green (high similarity) to red (low similarity), with the numbers associated with each box indicating the percentage similarity. P-type T4SS-encoding plasmids are designated as Group 1 (**C**) or Group 2 (**D**).

Given the diverse host range of the P-type T4SS-encoding plasmids within the *Acinetobacter* genus, plasmid genetic diversity was subsequently investigated (Fig. 2A). The plasmids ranged in size from 32.8 to 61.2 kb, with a mean of 44.5 kb (Fig. 2B; Table S1). All plasmids with quantifiable sequencing data demonstrated a low GC content (mean 39.0%), ranging from 36.2% to 42.3% (Table S1), consistent with the established literature on *Acinetobacter* (50) and this subset of T4SS-encoding plasmids (38).

**Fig 3:**
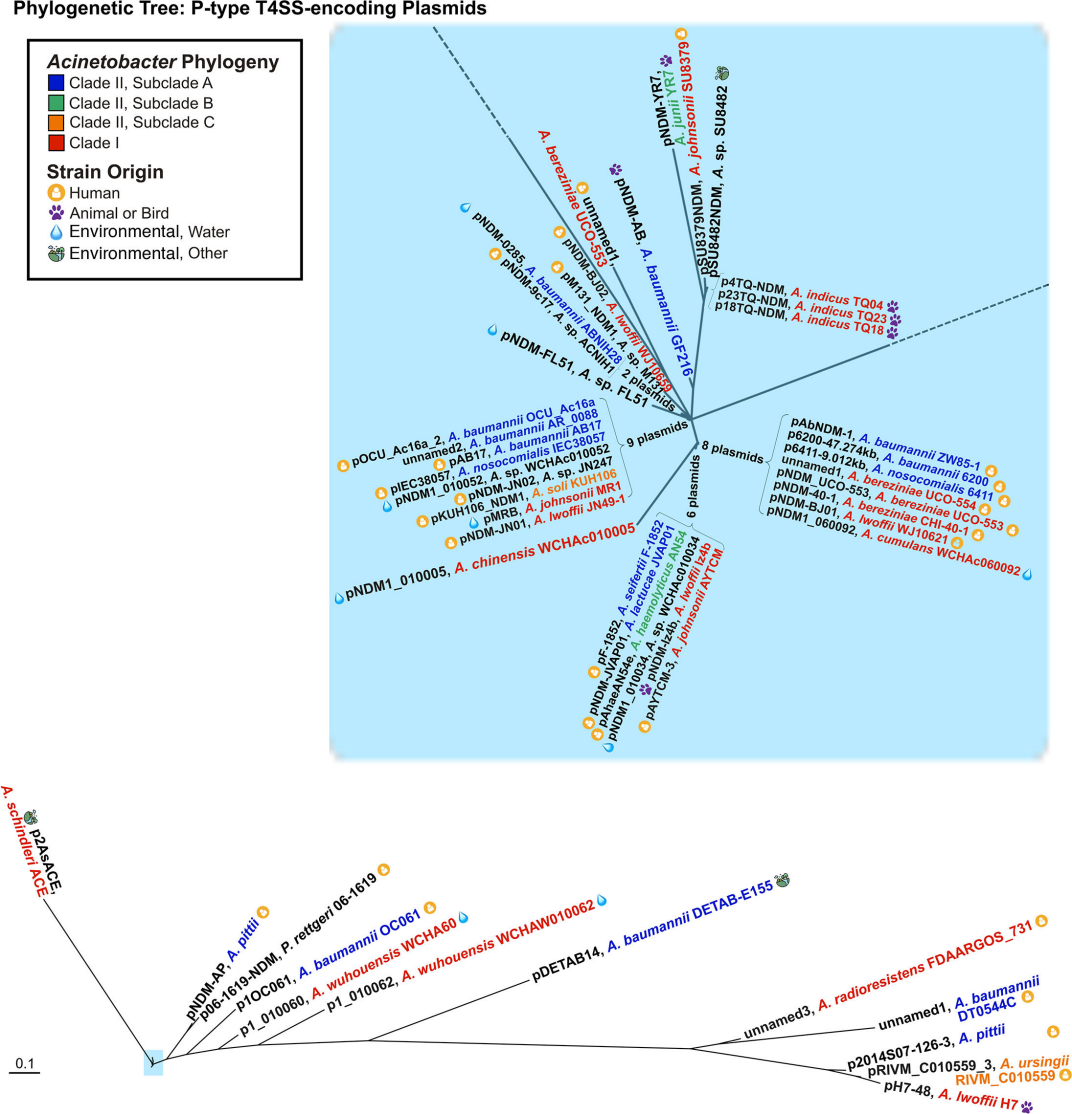
Phylogenetic analysis of T4SS-encoding plasmids. Phylogenetic tree of P-type T4SS-encoding plasmids with complete sequencing data. Where possible, host strains are color coded based on the previously established *Acinetobacter* clade (49), with subclades of Clade II designated as A to C. Strain origin is indicated based on the symbols shown in the legend. The region highlighted in light blue is shown in the expanded inset.

Phylogenomic analyses were subsequently performed using the software Gegenees (51). This genetic analysis indicated that the plasmids lay in two groups (Fig. 2C and D). The largest group of plasmids, denoted as Group 1, consisted of the majority of plasmids, representing 88% of plasmids assessed (Fig. 2C). This included all previously described P-type T4SS-encoding plasmids from the original study (38). The second smaller group of plasmids, denoted as Group 2, consisted of the remainder of strains (Fig. 2D). Within each group, plasmids demonstrated >70% coverage relative to the comparison plasmids pM131_NDM1 and p2014S07-126-3, respectively. Plasmids in Group 1 demonstrated <55% coverage relative to plasmids in Group 2. Importantly, both Group 1 and Group 2 plasmids were observed to be harbored in diverse *Acinetobacter* species, including *A. baumannii* but also spanning outside the clade associated with the ACB complex (20, 21) (Fig. 2C and D; Table S1). The phylogenomic designation into two groups observed by Gegenees was further supported by phylogenetic analyses (Fig. 3). Recently, new classification systems have been proposed for *Acinetobacter* plasmids (52, 53). In the context of *Acinetobacter* plasmids, the plasmid pNDM-BJ01 from this study was classified as a Group III-1b plasmid, harboring a MOB_Q1_ and no identifiable replicase gene (52). Consistent with this, 96% of plasmids demonstrated evidence of a relaxase or candidate pseudogene from the *mob_Q1_* family and no detectable replicase (Table S1).

For additional insight on plasmid divergence at the level of genetic rearrangements, the bioinformatics software Mauve (54) was used to perform a comparative genetic analysis of the P-type T4SS-encoding plasmids (Fig. S1 to S4). This software identifies conserved regions among sequences called locally collinear blocks, which are derived from whole genome or plasmid alignment analyses (54). As such, it allows for the detection of regions of genetic divergence such as insertions or deletions. Consistent with the analyses in Fig. 2C and D, Mauve analysis showed a large proportion of plasmids with high levels of conservation to the comparison plasmid pM131_NDM1 across the entirety of the sequence (Fig. S1 to S4). In addition, evidence of genetic rearrangements was also observed, both within and outside the P-type conjugative operon. For example, plasmid pRIVM_C010559_3 from *A. ursingii* RIVM_C010559 demonstrated a candidate insertion within the P-type conjugative operon (Fig. S4). The plasmid p2AsACE from *A. schindleri* ACE showed a large candidate insertion downstream of the P-type conjugative operon that included a region identified as unique among the plasmids in this study (Fig. S4). Genetic elements commonly identified within these regions included those annotated as mobile genetic elements and antibiotic resistance genes. For example, plasmid p1OC061 from *A. baumannii* OC061 harbored a candidate insertion within the P-type conjugative operon that included putative transposase genes and a large candidate insertion upstream of the operon harboring antibiotic resistance determinants (Fig. S3). Collectively, this suggests that genetic divergence exists among the P-type T4SS-encoding plasmids.

### P-type T4SS-encoding region: comparative analyses

To investigate the candidate P-type T4SS-encoding regions of the plasmids identified, blast analyses were performed against the previously established T4SS-associated proteins (38). From a genetic perspective, the original subset of plasmids was previously shown to harbor a core P-type conjugative operon encoding a series of *virB* genes, in addition to several other T4SS-associated genes (*traA, traC, traD,* and *trbN*) (38). Across the 53 plasmids, the majority of T4SS-associated genes detected were observed to be highly conserved (Table S2). Assessment of the P-type conjugative operons indicated that the region between *virB6* and *virB5* was exactly 13,295 bp in 77% of plasmids, with 91% of plasmids demonstrating a corresponding region within 100 bp of this (Table S2). Across all plasmids with complete sequencing data of this region, the P-type conjugative operon region ranged from 12,560 to 23,969 bp (Table S2).

**Fig 4:**
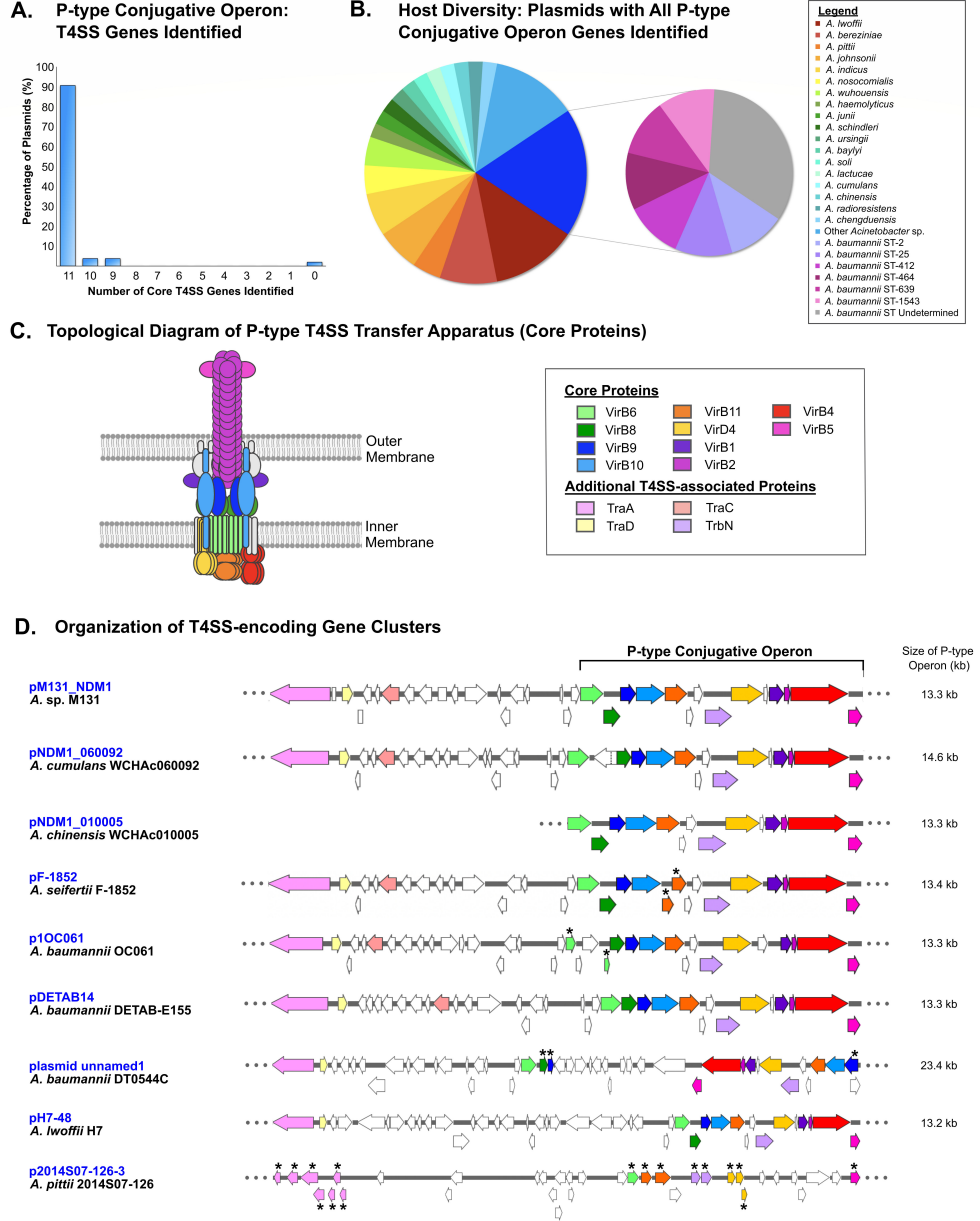
Diversity of P-type T4SS gene cluster in *Acinetobacter* plasmids. (**A**) Number of intact genes identified within the P-type conjugative operon, plotted as a bar graph. A total of 11 genes were assessed. (**B**) Phylogenetic classification of strains with plasmids harboring all P-type conjugative operon genes, shown as a pie chart. (**C**) A topological representation of the P-type T4SS transfer apparatus, based on the predicted structure (33, 55). Proteins with established positions within the three-dimensional structure are shown (designated as core proteins), and additional T4SS-associated proteins with contributions that have yet to be established are shown in the legend. (**D**) Linear gene map of P-type conjugative operon gene organization. The previously established cluster in pM131_NDM1 (38) is indicated as a comparison, with the additional T4SS-associated genes shown relative to the P-type conjugative operon. Linear gene maps of plasmids from this study indicate all open reading frames within the regions, with the size of the P-type conjugative operon shown to the right. T4SS-associated genes are color coded based on the legend shown in panel **C**, with all other open reading frames indicated in white. Asterisks (*) denote candidate pseudogenes.

At the amino acid level, T4SS-associated proteins were also observed to be highly conserved (Table S2), with 84% of proteins identified as 100% identical to the query amino acid sequence and 90% of proteins ≥98% identical (Table S2). In 91% of plasmids, genes encoding all 11 core proteins queried were identified (Fig. 4A; Table S2), with 81% of plasmids encoding all 14 T4SS-associated proteins queried (Table S2). With regard to host strain distribution, plasmids harboring all 11 core genes assessed were observed in the majority of *Acinetobacter* species identified and most of the *A. baumannii* sequence types (Fig. 4B).

For visualization of the T4SS-associated genes identified, linear gene maps were constructed. With respect to relative gene organization, the overall organization of the P-type conjugative operon and additional T4SS-associated genes was highly conserved across the majority of the 53 plasmids (Table S2). However, several plasmids were identified with regions that represented genetic divergence. For example, within the P-type conjugative operon of plasmid pNDM1_060092 from *A. cumulans* WCHAc060092, an additional region was identified between *virB6* and *virB8* not present in other plasmids (Fig. 4C and D; Table S2). This region harbored a transposase pseudogene. Similarly, within the additional T4SS gene-encoding region, plasmid pDETAB14 from *A. baumannii* DETAB-E155 harbored a region between *traD* and *traC* that might represent a genetic acquisition (Fig. 4D; Table S2). This region harbored genes annotated as encoding hypothetical proteins.

Several plasmids harbored one or more pseudogenes within the P-type conjugative operon, such as *virB11* in pF-1852 from *A. seifertii* F-1852 and *virB6* in p1OC061 from *A. baumannii* OC061 (Fig. 4D; Table S2). In addition, several plasmids demonstrated more extensive genetic variation. For example, plasmid unnamed1 from *A. baumannii* DT0544C contained a large region within the P-type conjugative operon that was inverted relative to the other plasmids, downstream of *virB6* (Fig. 4D; Table S2). Another striking example of genetic variation was plasmid p2014S07-126-3 from *A. pittii* 2014S07-126, which harbored regions with sequence similarity to the P-type conjugative operon and the additional T4SS gene encoding region in pM131_NDM1. However, the corresponding regions in p2014S07-126-3 harbored a series of gene remnants and pseudogenes, such that no intact T4SS-associated genes appeared to remain (Fig. 4D; Table S2). Collectively, this demonstrates that while overall the 53 plasmids identified harbored high levels of conservation with respect to the P-type conjugative operon and additional T4SS-associated genes, genetic diversity was present among the plasmids.

### P-type T4SS-encoding plasmids harbor diverse antibiotic resistance genes

**Fig 5:**
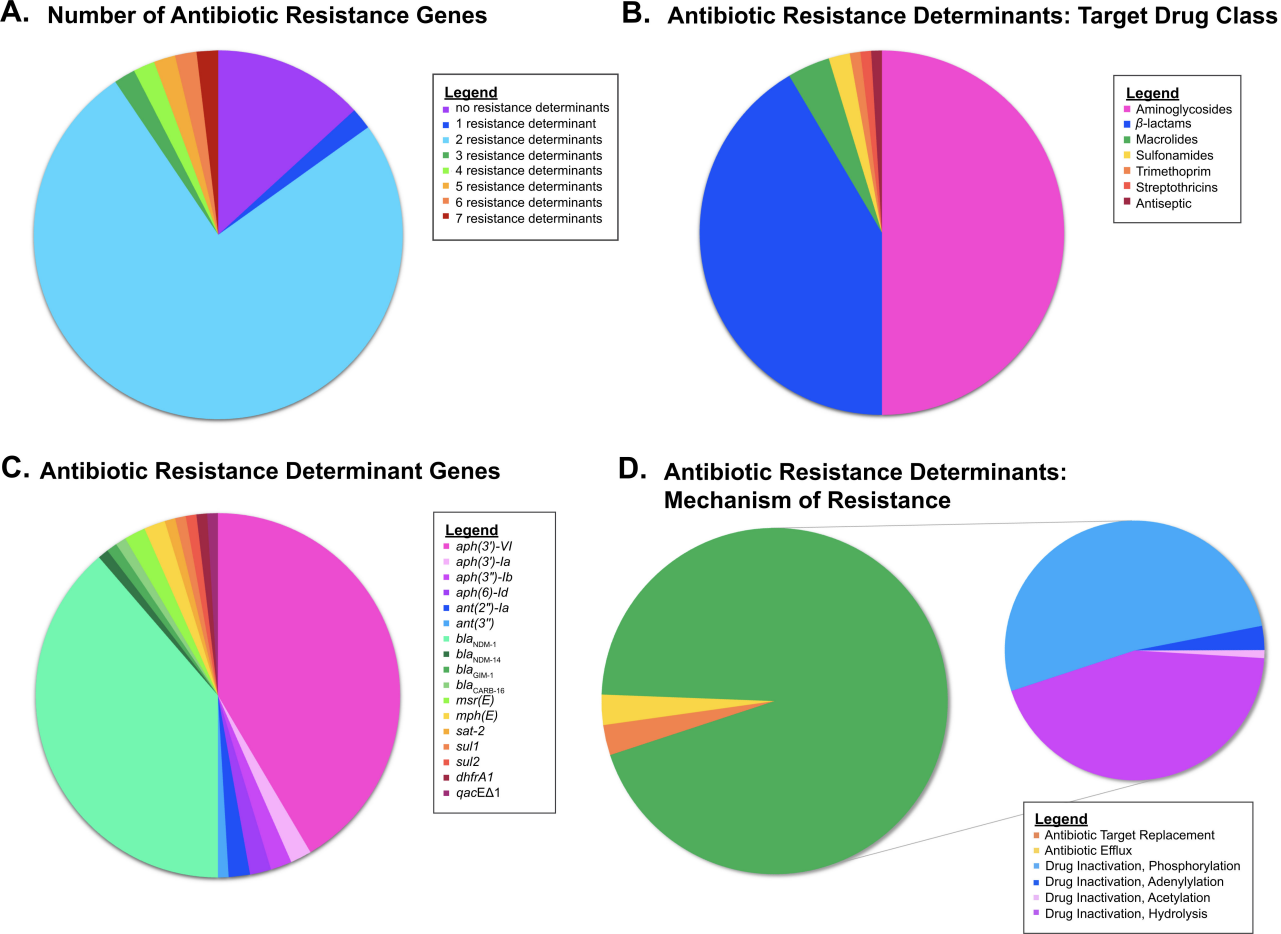
Antibiotic resistance determinants in P-type T4SS-encoding plasmids. (**A**) Frequency of resistance gene identification, shown as a pie chart. (**B**) Target drug class of resistance determinants, shown as a pie chart. (**C**) Distribution of antibiotic resistance determinants identified, shown as a pie chart. (**D**) Antibiotic resistance mechanism of the determinants identified, shown as a pie chart.

The spread of antibiotic resistance determinants, both clinically and in the environment, has been recognized by the WHO as an important global challenge necessitating a One Health perspective to understanding resistance (56, 57). Given the importance of understanding this reservoir among the *Acinetobacter* genus, the diversity of antibiotic resistance determinants harbored on the plasmids was examined. The original subset of P-type T4SS-encoding plasmids was characterized in the context of carbapenem resistance due to the presence of the *bla*_NDM-1_ resistance determinant, harbored on a mobile genetic element that also contains the aminoglycoside resistance gene *aph(3')-VI* (38). To assess the diversity of resistance genes harbored on the P-type T4SS-encoding plasmids, a systematic analysis was performed using the databases Comprehensive Antibiotic Resistance Database (CARD) (58) and ResFinder 4.1 (59). Analysis of the 53 plasmids uncovered a multitude of resistance determinants (Fig. 5; Table S3). In total, 87% of plasmids contained resistance genes (Fig. 5A), with 76% of plasmids harboring two resistance genes, consistent with the original study (38). The remaining plasmids were observed to harbor up to seven resistance determinants (Fig. 5A), with 9% of plasmids containing more than two resistance genes. Strains harboring more than two resistance genes included *A. baumannii, A. johnsonii*, and *A. schindleri*.

Resistance genes associated with aminoglycoside and β-lactam antibiotics represented the largest proportions, consistent with the original study (38); however, additional resistance determinants associated with these antibiotic classes were observed. Genes associated with resistance to macrolides, sulfonamides, trimethoprim, streptothricin, and antiseptics were also identified (Fig. 5B). In total, 17 different resistance genes were identified among the P-type T4SS-encoding plasmids spanning seven drug classes (Fig. 5B and C). This included aminoglycoside phosphotransferases that modify antibiotics at three different sites *(aph(3')-VI*, *aph(3')-Ia*, *aph(3″)-Ib,* and *aph(6)-Id*), aminoglycoside nucleotidyltransferases that modify at two different sites *(ant(2″)-Ia* and *ant(3*″)), and four different β-lactamases (*bla*_NDM-1_, *bla*_NDM-14_, *bla*_GIM-1_, and *bla*_CARB-16_). With respect to macrolides, resistance determinants associated with drug inactivation by phosphorylation (*mph(E*)) and antibiotic efflux (*msr(E*)) were observed, in addition to two different sulfamethoxazole resistance genes (*sul1* and *sul2*) (Fig. 5C). Resistance genes were also identified for trimethoprim (*dfrA1*), streptothricin (*sat-2*), and antiseptics (*qacE*Δ*1*) (Fig. 5C).

With respect to the mode of action associated with the resistance genes identified, enzymatic antibiotic inactivation represented 95% of genes, spanning 12 different determinants (Fig. 5D). Antibiotic efflux and drug target replacement were also modes of action associated with genes harbored on the P-type T4SS-encoding plasmids, representing 3% each (Fig. 5D).

**Fig 6:**
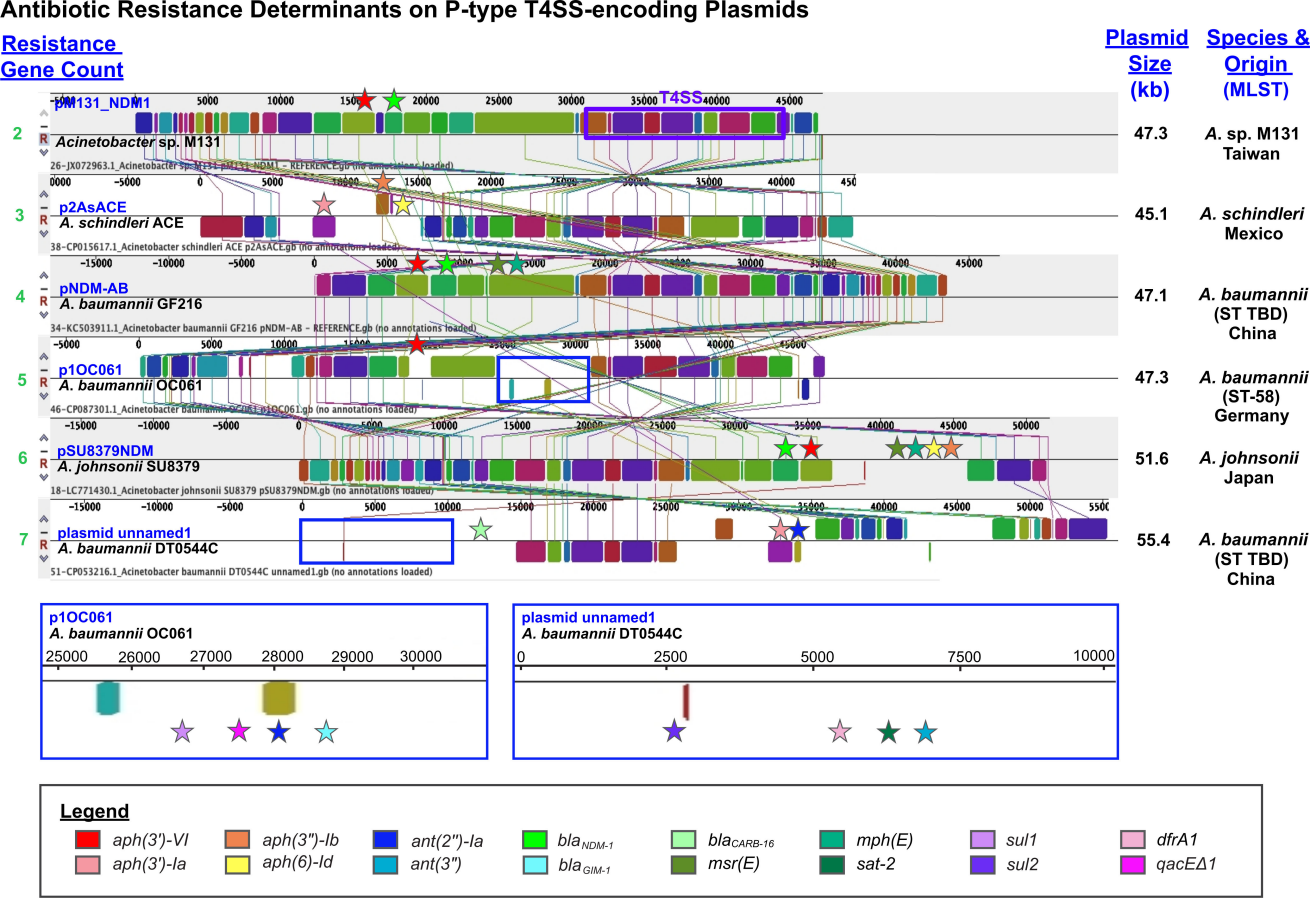
Antibiotic resistance genes in P-type T4SS-encoding plasmids. Multiple whole plasmid alignments for selected plasmids are shown, outlining regions of genetic rearrangements. Locally collinear blocks are indicated by Mauve in the same color. The P-type conjugative operon in reference plasmid pM131_NDM1 (38) is shown by a purple box. Antibiotic resistance genes are denoted at the associated location by stars, where the color of each star is reflected in the legend.

Across the 46 plasmids harboring resistance determinants, the relative positioning of the *aph(3')-VI* and *bla*_NDM-1_ genes was observed to be conserved (Fig. 6; Table S3), consistent with the literature (38). However, the *aph(3')-Ia*, *aph(3″)-Ib*, *aph(6)-Id,* and *ant(2″)-Ia* genes were identified at more than one unique location (Fig. 6). Interestingly, the aminoglycoside resistance genes *mph(E*) and *msr(E*) were always observed in tandem, either upstream or downstream of the region harboring *aph(3')-VI* and *bla*_NDM-1_ (Fig. 6). Collectively, this demonstrates *Acinetobacter*’s capacity for genetic plasticity, and in particular, the acquisition of antibiotic resistance genes.

Horizontal gene transfer plays an intrinsic role in pathogen evolution and is often facilitated by the presence of mobile genetic elements such as insertion sequences (60). To provide further insight into the context of genetic diversity, the P-type T4SS-encoding plasmids were examined for the presence of candidate mobile genetic elements using MobileElementFinder (61). Candidate insertion sequences were identified in 98% of plasmids (Table S4). With regard to antibiotic resistance determinants, the vast majority of resistance genes were determined to be within 1.2 kb of candidate insertion sequences (Tables S3 and S4). This suggests the importance of insertion sequences in the evolution of these T4SS-encoding plasmids.

## DISCUSSION

Bacterial P-type T4SSs play a central role in microbial adaptation and evolution, contributing to the spread of genetic material among populations, as well as the secretion of proteins such as virulence factors and small molecules that mediate host-microbe interactions and microbe-microbe interactions to promote bacterial survival (62). The WHO and CDC have collectively recognized *A. baumannii* as a priority pathogen of special importance for the research community, partly due to its capacity for horizontal gene transfer that has resulted in the evolution of MDR and XDR strains (3). Increasing acknowledgment that diverse *Acinetobacter* species both inside and outside the ACB complex, including several emerging pathogens, play important roles in human disease (12, 13, 20–26) is highlighting the greater need to understand pathogen evolution and molecular determinants that contribute to infection.

Here, we performed a systematic analysis to investigate the genetic diversity and geographical distribution of a group of P-type T4SS-encoding plasmids in *Acinetobacter* previously highlighted for the capacity to confer resistance to carbapenem and aminoglycoside antibiotics and for their transmissibility among *Acinetobacter* (38–46). We characterize a group of 53 plasmids from 20 defined species of *Acinetobacter* including five species within the established pathogen group ACB complex (*A. baumannii A. pittii*, *A. nosocomialis*, and *A. seifertii*) and species outside the ACB complex that are established to be associated with human disease (*A. lwoffii*, *A. johnsonii, A. haemolyticus*, *A. schindleri, A. ursingii, A. junii, A. soli, A. bereziniae,* and *A. radioresistens*) (13, 23–26), several of which are considered to be emerging pathogens (24–26, 63). These species represented the majority of strains harboring P-type T4SS-encoding plasmids, comprising 70% of strains. Of importance, *A. baumannii* represented the largest proportion of P-type T4SS-encoding plasmid host strains (21% of strains). Among these, this study identified T4SS-encoding plasmids in a number of *Acinetobacter* species not previously reported to harbor this group of plasmids, such as *A. seifertii, A. radioresistens,* and *A. baylyi*. Collectively, this demonstrates that P-type T4SS-encoding plasmids are circulating in more diverse strains of *Acinetobacter* than previously appreciated.

Of special note, this study also uncovered a T4SS-encoding plasmid from *Providencia rettgeri* (plasmid p06-1619-NDM). This plasmid genetically clustered with Group 1 plasmids (Fig. 2C) and demonstrated 84% sequence identity to four *Acinetobacter* plasmids (pNDM-0285 from *A. baumannii* ABNIH28, pNDM-AP from *A. pittii*, pNDM1_010052 from *A*. sp. WCHAc010052, and pNDM-9c17 from *A*. sp. ACNIH1). Analysis of the associated genome data indicated that the strain does belong to the *Providencia* genus, consistent with the original report of this strain (64). Of importance, this MDR plasmid harbored both β-lactam and aminoglycoside resistance genes (*bla*_NDM-1_ and *aph(3')-VI,* respectively) common across the majority of the *Acinetobacter* plasmids studied and was associated with a hospital-associated outbreak in Mexico (64). This highlights the potential for these determinants to transfer to other clinical pathogens.

The T4SS-encoding plasmids characterized in this study demonstrated exceptional conservation in the P-type conjugative operon, such that 91% of plasmids encoded full-length proteins for all core genes examined (Fig. 4A) and 84% of proteins identified demonstrated 100% identity to the query sequence. In the context of horizontal gene transfer, many members of this group of P-type T4SS-encoding plasmids have been established to be transmissible (38–46). Therefore, this high level of conservation may be suggestive of an evolutionarily important role in microbial fitness. It is thus possible that the multidrug resistance phenotype associated with the majority of plasmids (harboring *bla*_NDM-1_ and *aph(3')-VI*) may contribute to fitness in clinical settings where antimicrobial treatment is required. It is also possible that other genes harbored on the plasmids may contribute to fitness. In the context of protein secretion, P-type T4SS-mediated virulence factor secretion can contribute to diverse host-pathogen interactions beneficial to pathogen success such as the induction of pathogen uptake into host cells, manipulation of host cell growth and/or integrity, and intracellular trafficking (35–37). Given that *Acinetobacter* plasmid T4SS proteins demonstrate sequence similarity to P-type T4SSs in pathogens that secrete virulence factors (35–37), it is possible that the T4SS can also mediate the delivery of proteins that mediate pathogenesis.

While overall an exceptional level of conservation was observed across T4SS-associated proteins encoded in the P-type conjugative operon, this study also identified interesting examples of genetic diversity among the plasmids. This included candidate insertions, genetic rearrangements, pseudogenes, and regions with many gene remnants. Of interest to note, many regions with candidate insertions were observed to harbor proteins of no established function (annotated as hypothetical proteins). In the future, it would be interesting to investigate whether these genes could contribute to microbial fitness.

*Acinetobacter* is acknowledged by both the WHO and CDC as a pathogen of high clinical importance in large part due to its capacity for acquiring antibiotic resistance genes to important clinical therapeutics, such as carbapenems, which has resulted in the evolution of MDR and XDR lineages (3, 4). An analysis of the antibiotic resistome of the P-type T4SS-encoding plasmids uncovered an unexpected level of diversity of resistance genes, with 17 different genes spanning seven drug classes identified across the 53 plasmids (Fig. 5B and C). Given that 87% of plasmids harbored resistance genes, that the majority of plasmids were considered to be MDR (up to seven resistance genes), and that transmissibility has been established for many of the plasmids (38–46), these observations were of special concern. Of importance to note were the number of different resistance genes identified to the clinically important drug classes aminoglycosides and β-lactams. Across the plasmids, six different aminoglycoside resistance genes (*aph(3')-VI*, *aph(3')-Ia*, *aph(3″)-Ib*, *aph (6)-Id*, *ant(2″)-Ia,* and *ant(3*″)) and four different β-lactam resistance determinants (*bla*_NDM-1_, *bla*_NDM-14_, *bla*_GIM-1_, and *bla*_CARB-16_) were observed. As aminoglycosides and β-lactams are drug classes of importance to the healthcare community, these resistance determinants will be important to monitor in the future.

The *Acinetobacter* genus, including *A. baumannii*, is capable of occupying diverse ecological niches outside of human infection, including water and soil ecosystems, and animal-, plant-, and insect-associated settings (13–15). Diverse *Acinetobacter* species have also been established as problematic animal and fish pathogens (27–30), where several have been associated with multi-drug-resistant infections (28, 65, 66). In this study, strains harboring T4SS-encoding plasmids were isolated from animals (cow, pig, chicken, and cat) and environmental settings, and additional antibiotic resistance genes beyond *bla*_NDM-1_ and *aph(3')-VI* were identified. For example, plasmid pNDM-AB from *A. baumannii* GF216, derived from an infection in a pig, harbored *bla*_NDM-1_ and *aph(3')-VI* in addition to macrolide resistance genes *msr(E*) and *mph(E*), and the plasmid was also shown to be capable of transmission (46). Given that the majority of plasmids harbored multiple resistance genes, this plasmid family should also be of importance to consider for agricultural and environmental surveillance efforts.

Collectively, this study demonstrated that this group of P-type T4SS-encoding plasmids is larger and more widespread across the *Acinetobacter* genus than previously appreciated, including diverse species of clinical importance such as *A. baumannii*. The exceptional level of conservation of T4SS-associated proteins may suggest an important role the secretion system plays in microbial fitness, such as the phenotype of multidrug resistance associated with clinical settings. Given the diversity of resistance determinants identified on these P-type T4SS-encoding plasmids, they represent an important genetic element for future clinical monitoring. As incidences of infection with MDR and XDR strains increase globally, including other antibiotics of importance such as the last resort drug colistin (67), it is becoming increasingly important to consider non-traditional approaches to drug discovery. Secretion system inhibitors are an area of active interest in the antimicrobial research community, and unlike conventional antibiotics, treatment is associated with minimal selection for resistance-associated mutations (68). As such, inhibition of this *Acinetobacter* P-type T4SS may represent a valuable opportunity for exploration in the future.

## MATERIALS AND METHODS

### T4SS gene cluster plasmid identification and MLST analysis

T4SS-encoding plasmids with sequence similarity to the P-type T4SS gene cluster in pM131_NDM1 were identified using blastn (69). A bioinformatic analysis of the NCBI nucleotide collection was performed using blastn with the P-type conjugative operon from pM131_NDM1 (accession JX072963) (38) as the query and default parameters. Identity and coverage thresholds of 70% were used.

To assess *A. baumannii* host strain diversity, multilocus sequence type was determined using previously established approaches (48, 70). When sufficient genome sequencing data were available for strains of interest, accession numbers associated with strain genomes were submitted to PubMLST (https://pubmlst.org/) as queries. Typing was performed using the established MLST Pasteur and Oxford schemes for *A. baumannii* (48, 70).

### Replicase and relaxase analyses

A previously established reference list of *Acinetobacter* replicases (53) was used for the identification of candidate replicase genes in T4SS-encoding plasmids. Blastn analyses (69) were performed against the reference replicases using plasmid accession numbers as queries. Similarly, previously established reference mobilization proteins (relaxases) have been established for plasmid characterization (52, 71). Blastx analyses were performed against the reference replicases using plasmid accession numbers as queries (52, 71). Open reading frames were manually curated using a coverage threshold of 80% (72, 73), and for unannotated plasmids, candidate open reading frames were assessed with the software SnapGene (www.snapgene.com).

### Gegenees plasmid analysis

Phylogenomic analyses of the P-type T4SS-encoding plasmids were performed using the software Gegenees (51). Full-length GenBank DNA sequences were submitted for Gegenees analysis using the recommended parameters for shorter sequences (blastn, fragment size 200, step size 100) (51).

The similarity matrix was shown as a heat map. Plasmids denoted as Group 1 demonstrated <55% coverage to plasmids in Group 2, and within each group, plasmids demonstrated >70% coverage relative to the comparison plasmids pM131_NDM1 and p2014S07-126-3, respectively.

### Phylogenetic analyses

Phylogenetic analyses were performed with complete sequences of P-type T4SS-encoding plasmids. Full-length sequences were aligned using ClustalW (74), and phylogenetic tree construction was performed with MrBayes (75), using 200,000 generations sampled every 100 generations, with a gamma distribution model and invariant class. Visualization of the phylogenetic tree was performed with the tool Interactive Tree Of Life (76).

### Mauve plasmid analysis

Comparative genetic analyses were performed using the bioinformatics software Mauve (54), which compares sequences at the level of genetic rearrangements. ProgressiveMauve (https://darlinglab.org/mauve) was used to perform alignments with the recommended default parameters and default HOXD scoring matrix. The plasmid sequence for pM131_NDM1 was used as a reference. The recommended seed families parameter was selected, as it has been shown to improve anchoring sensitivity in regions below 70% identity (54).

### P-type T4SS gene cluster analysis

The previous study described a P-type conjugative operon in addition to several other T4SS-associated genes in eight *Acinetobacter* plasmids (pM131_NDM1, pMS32-2, pAP_D499, pNDM-BJ01, pNDM-BJ02, pAbNDM-1, pNDM-AB, and pNDM-BJ01) (38). To identify and assess individual candidate T4SS-encoding genes, blast analyses were performed (69). Amino acid sequences (VirB6, VirB8, VirB9, VirB10, VirB11, TrbN, VirD4, VirB1,VirB2, VirB4, VirB5, TraA, TraD, and TraC) from the plasmid pM131_NDM1 (accession JX072963.1) (38) were used as queries, as previously reported in the original study (38). Many of the plasmids identified consisted of unannotated and untranslated regions. As such, tblastn was used to allow for a systematic analysis across all plasmid sequences. Similar to blastp, Tblastn quantifies amino acid sequence conservation. All open reading frames identified were manually curated using a coverage threshold of 80% (72, 73). An internal analysis could not verify the genes previously reported as *virC1* and *virB7* (38), presumably due to genetic divergence from previously characterized proteins. As a result, these were excluded from the analysis. Where the plasmid sequences were unannotated, candidate open reading frames were curated using SnapGene.

### Antibiotic resistance determinant analysis

CARD (58) and ResFinder 4.1 (59) were used to identify candidate antibiotic resistance determinants. For CARD version 3.1.0 (https://card.mcmaster.ca/), the Resistance Gene Identifier tool was used, where plasmid accession numbers were submitted using RGI Criteria thresholds Perfect and Strict. For ResFinder 4.1 (https://cge.cbs.dtu.dk/services/ResFinder/), plasmid sequences were submitted as fasta files. For the identification of resistance determinants, an identity threshold of 90% and a minimal length of 60% were used (77, 78). Subsequent to identification, each open reading frame was manually curated using a coverage threshold of 80% (72, 73).

### Mobile genetic elements analysis

The program MobileElementFinder (61) was used for the identification of candidate mobile genetic elements such as insertion sequences. With MobileElementFinder (https://cge.cbs.dtu.dk/services/MobileElementFinder/), sequences of plasmids were uploaded for analysis in .fasta format. The analysis was performed using recommended default parameters.

## SUPPLEMENTAL MATERIAL

Supplemental material aac.01038-24-s0001.pdf Figures S1 to S4; captions for Tables S1 to S4.

Table S1 aac.01038-24-s0002.xlsx Bacterial strains identified in this study.

Table S2 aac.01038-24-s0003.xlsx Candidate T4SS-encoding genes identified by Blast analysis.

Table S3 aac.01038-24-s0004.xlsx Antibiotic resistance determinants detected on T4SS-encoding plasmids.

Table S4 aac.01038-24-s0005.xlsx Mobile genetic elements detected on T4SS-encoding plasmids.

ASM does not own the copyrights to Supplemental Material that may be linked to, or accessed through, an article. The authors have granted ASM a non-exclusive, world-wide license to publish the Supplemental Material files. Please contact the corresponding author directly for reuse.
