# Supplementary material for: Analysis of Acinetobacter P-type type IV secretion system-encoding plasmid diversity uncovers extensive secretion system conservation and diverse antibiotic resistance determinants
Source: Antimicrob Agents Chemother. 2024 Nov 4;68(12):e01038-24. doi: 10.1128/aac.01038-24 (PMC11619351; doi:10.1128/aac.01038-24)
Supplement: Supplemental material — Figures S1 to S4; captions for Tables S1 to S4. [file aac.01038-24-s0001.pdf]

SUPPLEMENTAL MATERIAL

Analysis of *Acinetobacter* P-type Type IV Secretion System-encoding Plasmid Diversity Uncovers Extensive Secretion System Conservation and Diverse Antibiotic Resistance Determinants

Mosopefoluwa T. Oke[1,2], Kailey Martz[1,2†], Mădălina Mocăniță[1,2†], Sara Knezevic[1,2] and Vanessa M. D'Costa[1,2*]

[1] Department of Biochemistry, Microbiology and Immunology, University of Ottawa, Ottawa, Ontario, Canada, K1H 8M5
[2] Centre for Infection, Immunity and Inflammation, University of Ottawa, Ottawa, Canada, K1H 8M5

* Corresponding author:
Vanessa M. D'Costa
Department of Biochemistry, Microbiology and Immunology
University of Ottawa
451 Smyth Road
Ottawa, Ontario, Canada
K1H 8M5
Tel: (613)-562-2800 ext 4541
E-mail: vdcosta@uottawa.ca

† These authors contributed equally

SUPPLEMENTAL FIGURES

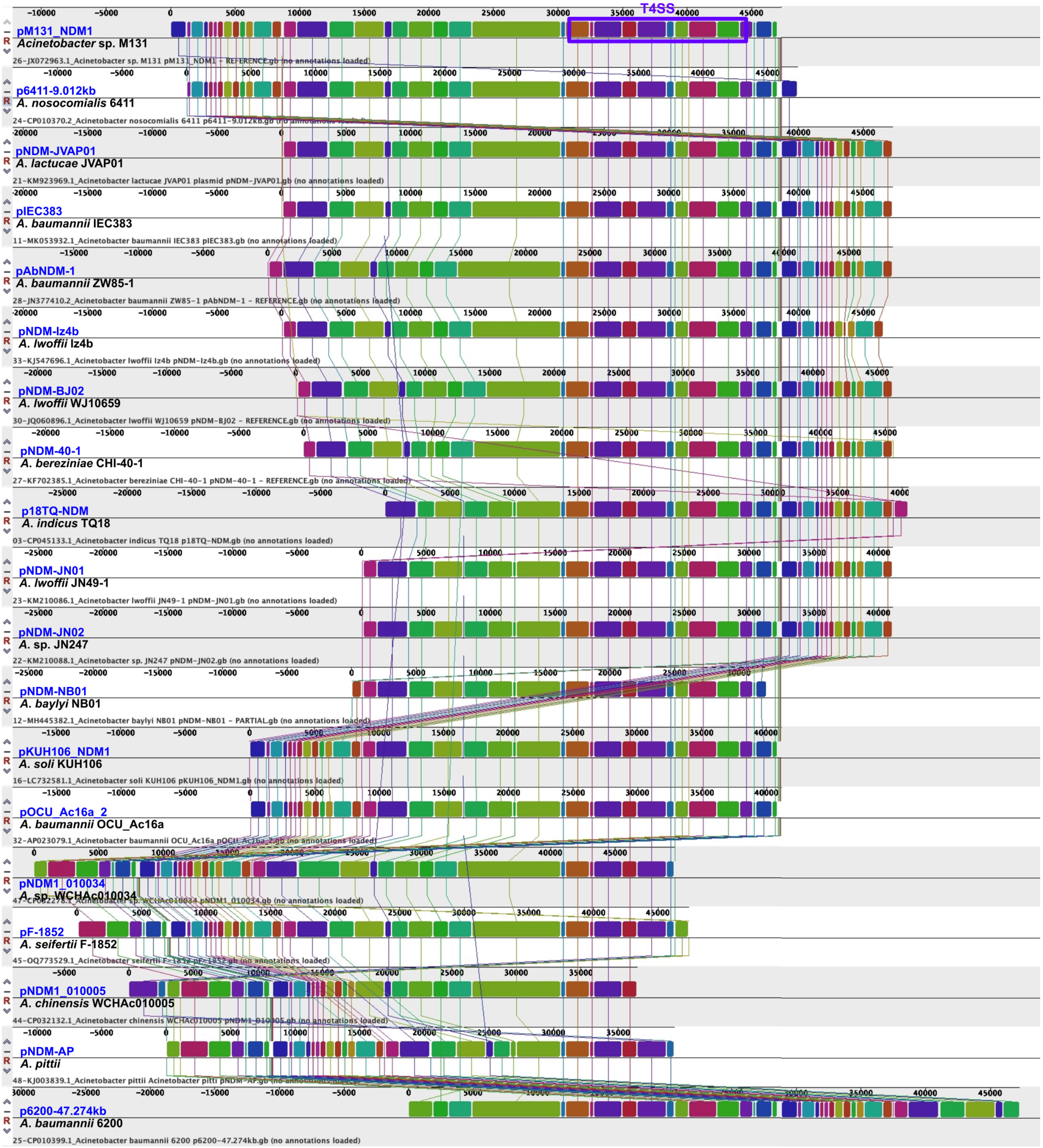

Figure S1. Mauve Analysis of P-type T4SS-encoding Plasmids. Multiple whole plasmid alignments were generated using the bioinformatics software Mauve, outlining regions of genetic rearrangements. Between plasmids, locally collinear blocks (LCBs) are denoted by Mauve in the same colour. The P-type conjugative operon in reference plasmid pM131_NDM1 is shown by a purple box, denoted as T4SS.

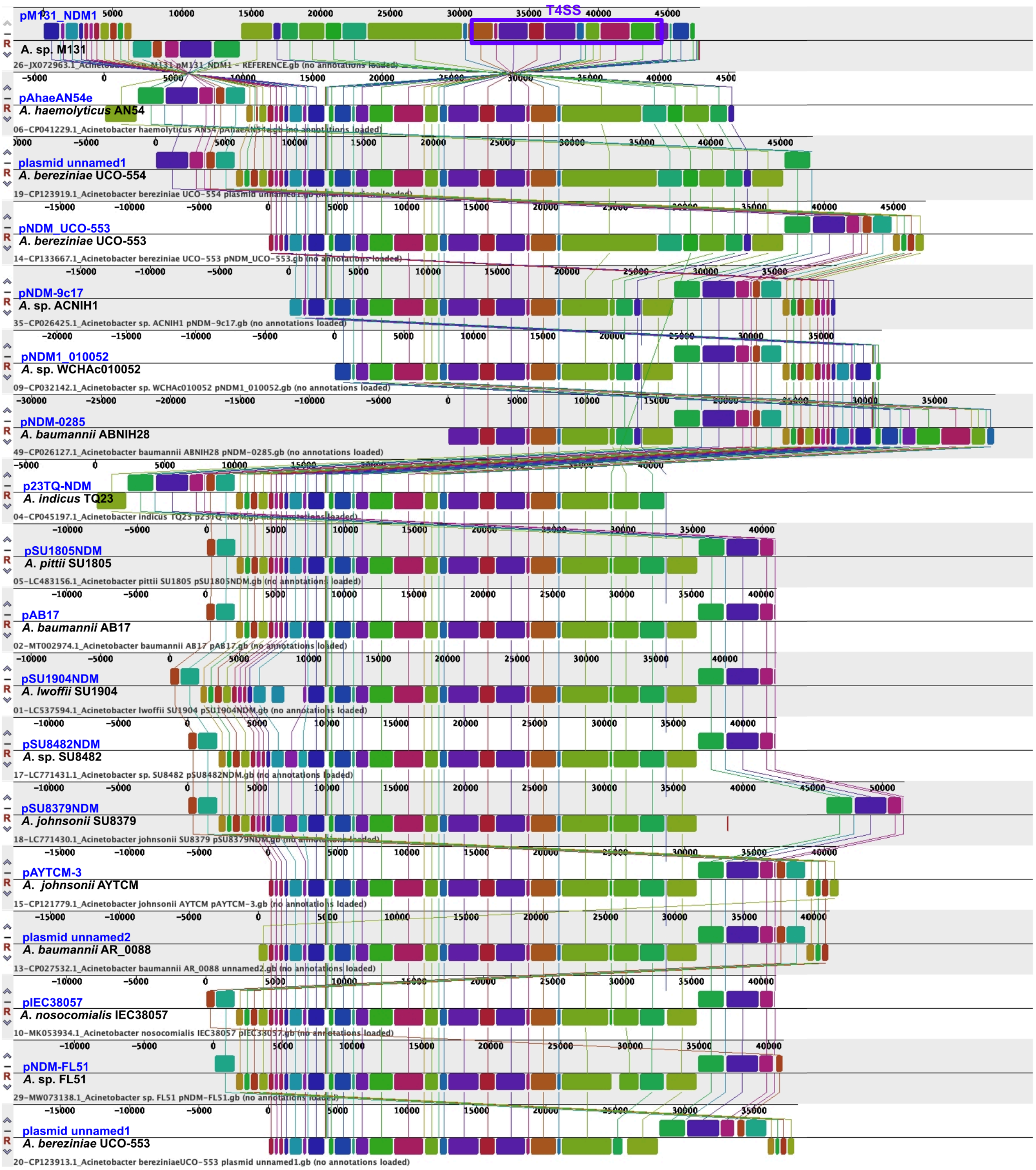

T4SS
pM131_NDM1
A. sp. M131
26-JX072963.1_Acinetobacter sp. M131 pM131_NDM1 - REFERENCE.gb (no annotations loaded)
pAhaeAN54e
A. haemolyticus AN54
06-CP041229.1_Acinetobacter haemolyticus AN54 pAhaeAN54e.gb (no annotations loaded)
plasmid unnamed1
A. bereziniae UCO-554
19-CP123919.1_Acinetobacter bereziniae UCO-554 plasmid unnamed.gb (no annotations loaded)
pNDM_UCO-553
A. bereziniae UCO-553
14-CP133667.1_Acinetobacter bereziniae UCO-553 pNDM_UCO-553.gb (no annotations loaded)
pNDM-9c17
A. sp. ACNIH1
35-CP026425.1_Acinetobacter sp. ACNIH1 pNDM-9c17.gb (no annotations loaded)
pNDM1_010052
A. sp. WCHAc010052
09-CP032142.1_Acinetobacter sp. WCHAc010052 pNDM1_010052.gb (no annotations loaded)
pNDM-0285
A. baumannii ABNIH28
49-CP026127.1_Acinetobacter baumannii ABNIH28 pNDM-0285.gb (no annotations loaded)
p23TQ-NDM
A. indicus TQ23
04-CP045197.1_Acinetobacter indicus TQ23 p23TQ-NDM.gb (no annotations loaded)
pSU1805NDM
A. pittii SU1805
05-LC483156.1_Acinetobacter pittii SU1805 pSU1805NDM.gb (no annotations loaded)
pAB17
A. baumannii AB17
02-MT002974.1_Acinetobacter baumannii AB17 pAB17.gb (no annotations loaded)
pSU1904NDM
A. lwoffii SU1904
01-LC537594.1_Acinetobacter lwoffii SU1904 pSU1904NDM.gb (no annotations loaded)
pSU8482NDM
A. sp. SU8482
17-LC771431.1_Acinetobacter sp. SU8482 pSU8482NDM.gb (no annotations loaded)
pSU8379NDM
A. johnsonii SU8379
18-LC771430.1_Acinetobacter johnsonii SU8379 pSU8379NDM.gb (no annotations loaded)
pAYTCM-3
A. johnsonii AYTCM
15-CP121779.1_Acinetobacter johnsonii AYTCM pAYTCM-3.gb (no annotations loaded)
plasmid unnamed2
A. baumannii AR_0088
13-CP027532.1_Acinetobacter baumannii AR_0088 unnamed2.gb (no annotations loaded)
pIEC38057
A. nosocomialis IEC38057
10-MK053934.1_Acinetobacter nosocomialis IEC38057 pIEC38057.gb (no annotations loaded)
pNDM-FL51
A. sp. FL51
29-MW073138.1_Acinetobacter sp. FL51 pNDM-FL51.gb (no annotations loaded)
plasmid unnamed1
A. bereziniae UCO-553
20-CP123913.1_Acinetobacter bereziniaeUCO-553 plasmid unnamed1.gb (no annotations loaded)

Figure S2. Mauve Analysis of P-type T4SS-encoding Plasmids. Multiple whole plasmid alignments were generated using the bioinformatics software Mauve, outlining regions of genetic rearrangements. Between plasmids, locally collinear blocks (LCBs) are denoted by Mauve in the same colour. The P-type conjugative operon in reference plasmid pM131_NDM1 is shown by a purple box, denoted as T4SS.

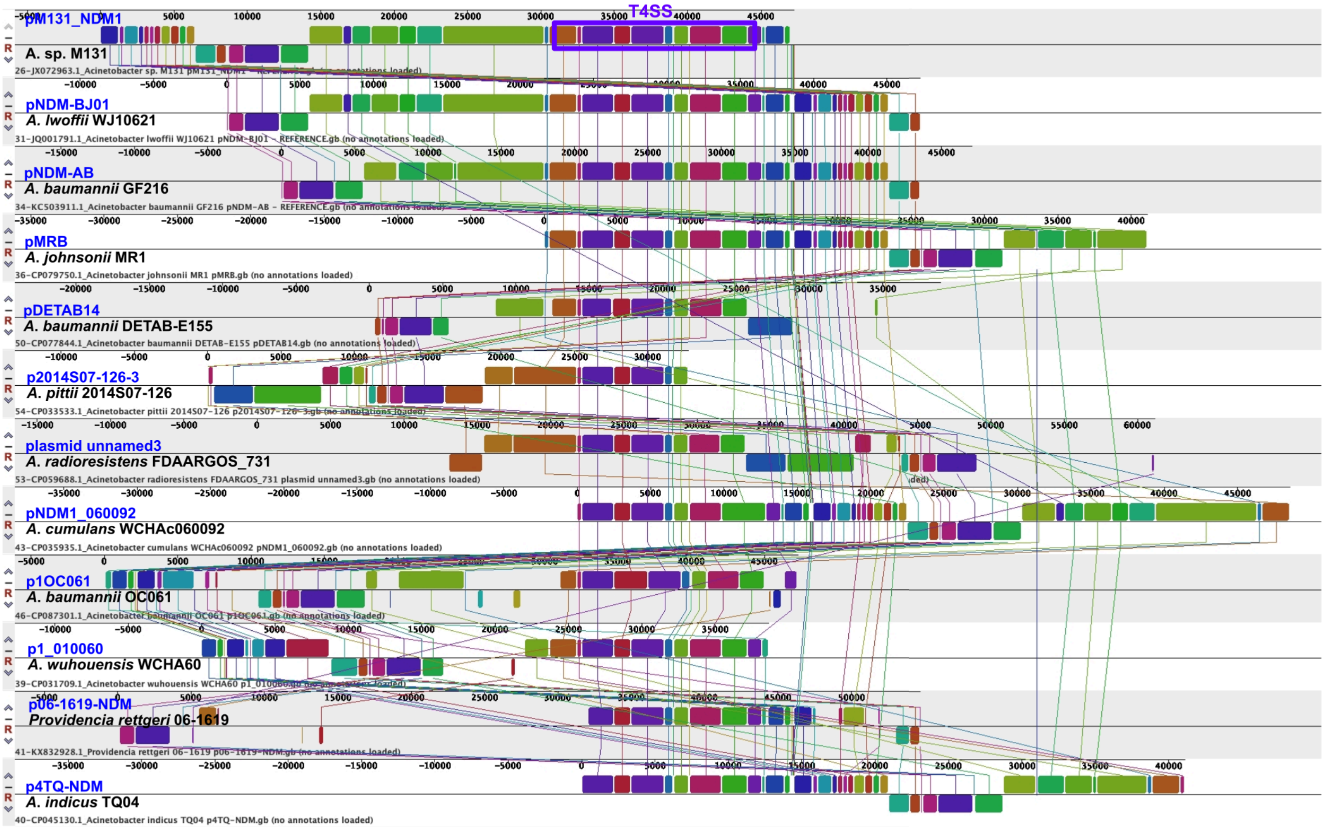

Figure S3. Mauve Analysis of P-type T4SS-encoding Plasmids. Multiple whole plasmid alignments were generated using the bioinformatics software Mauve, outlining regions of genetic rearrangements. Between plasmids, locally collinear blocks (LCBs) are denoted by Mauve in the same colour. The P-type conjugative operon in reference plasmid pM131_NDM1 is shown by a purple box, denoted as T4SS.

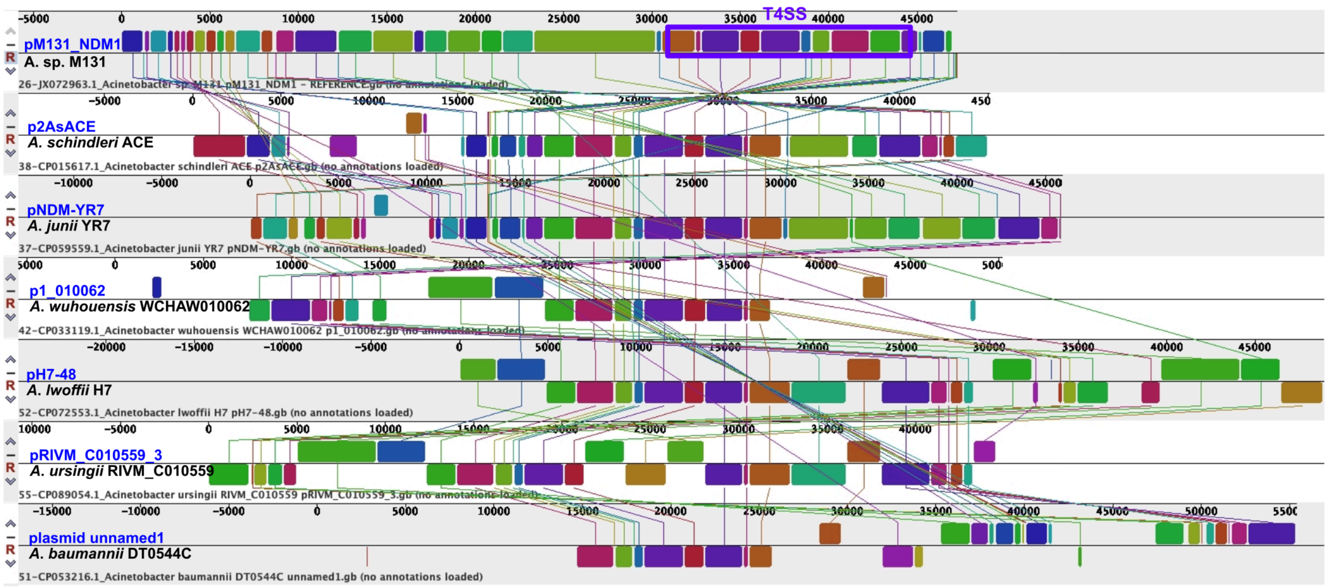

Figure S4. Mauve Analysis of P-type T4SS-encoding Plasmids. Multiple whole plasmid alignments were generated using the bioinformatics software Mauve, outlining regions of genetic rearrangements. Between plasmids, locally collinear blocks (LCBs) are denoted by Mauve in the same colour. The P-type conjugative operon in reference plasmid pM131_NDM1 is shown by a purple box, denoted as T4SS.

SUPPLEMENTAL TABLES

Note: Tables S1-S4 are included as separate files

Table S1. Bacterial Strains Identified in This Study

Table S2. Candidate T4SS-encoding Genes Identified by Blast Analysis

Table S3. Antibiotic Resistance Determinants Detected on T4SS-encoding Plasmids

Table S4. Mobile Genetic Elements Detected on T4SS-encoding Plasmids
